# Supplementary material for: Peroxisome Proliferator FpPEX11 Is Involved in the Development and Pathogenicity in Fusarium pseudograminearum
Source: Int J Mol Sci. 2022 Oct 12;23(20):12184. doi: 10.3390/ijms232012184 (PMC9603656; doi:10.3390/ijms232012184)
Supplement: Supplementary file 1 [file ijms-23-12184-s001.zip › ijms-1933254-supplementary.pdf]

International Journal of
Molecular Sciences

MDPI

Supplementary Materials

Peroxisome Proliferator FpPEX11 Is Involved in the Development and Pathogenicity in *Fusarium pseudograminearum*

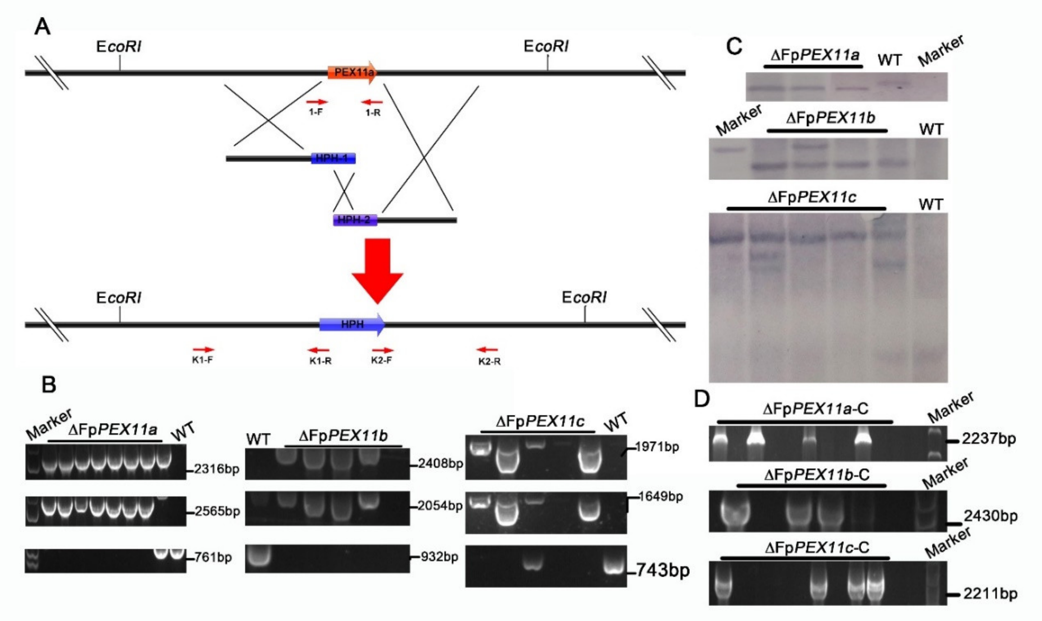

Figure S1. Construction of Δ*FpPEX11* and complementary strains in *F. pseudograminearum.* (**A**) Gene deletion strategies; (**B**) PCR confirmation; (**C**) Southern Blot; (**D**) Complemented strains of PCR confirmation.

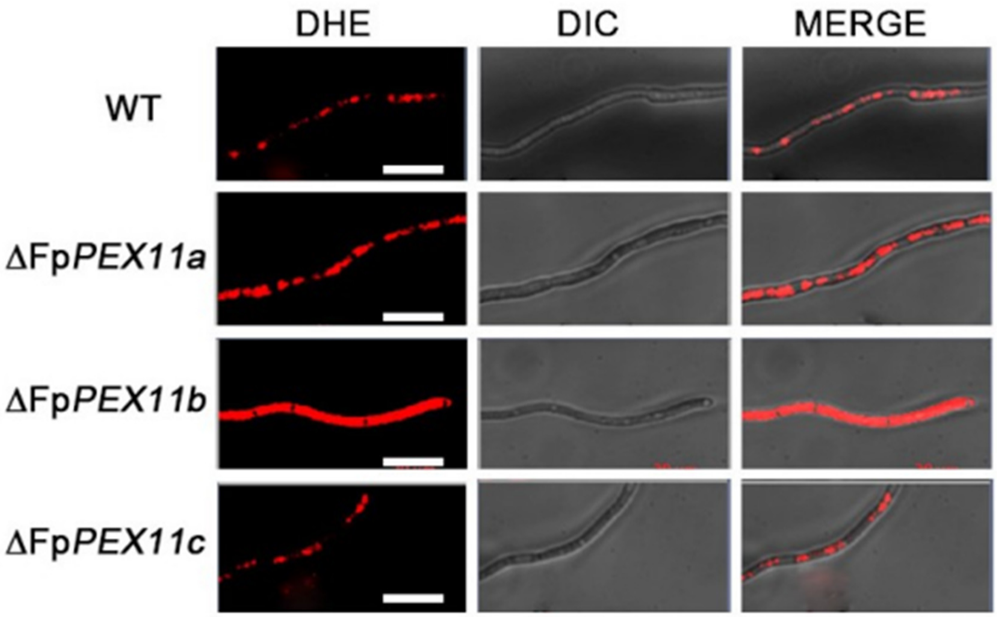

Figure S2. Δ*FpPEX11* promotes ROS accumulation. Fungal hyphae were stained with dihydroethidium (DHE) to visualize ROS in cells. Scale bar = 20 µm.

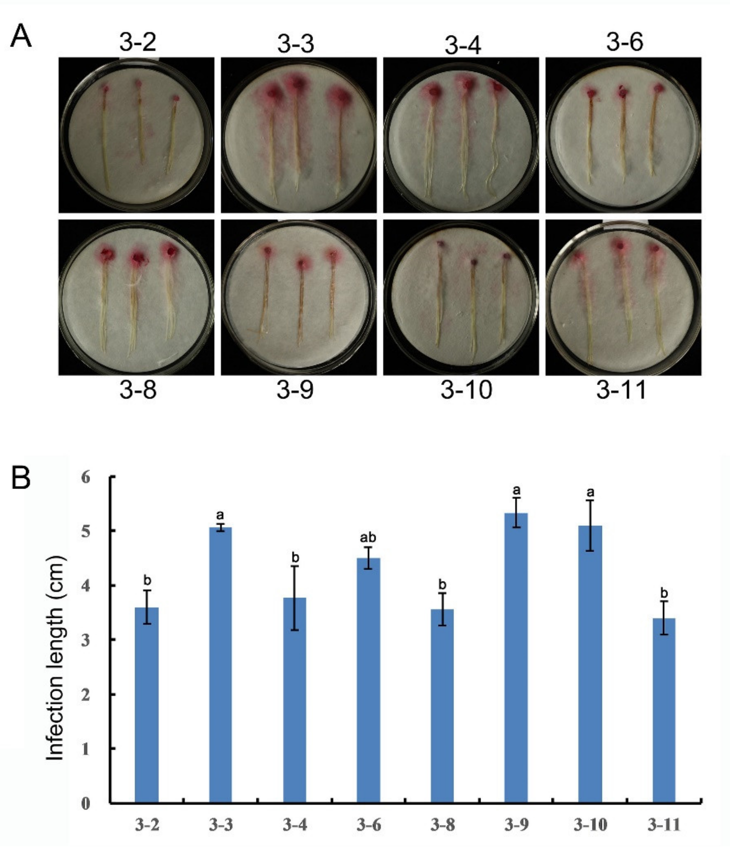

Figure S3. Pathogenicity of candidate strains in *F. pseudograminearum*. (**A**) Infestation of corn whiskers 5 d after inoculation; (**B**) Determination of the length of infestation of corn whiskers.

Table S1. Primers used in this study.

引物<br>**Primer**	引物序列<br>**Sequence(5′-3′)**	片段描述<br>**Relevant characteristics**
a-AF	GAATGTCTTTGCCTCGTATC	5′-flanking of *FpPEX11a* gene(1033bp)
a-AR	TTGACCTCCACTAGCTCCAGCCAAGATGGCGATGGAAA-TAGAGC	
a-BF	GAAATGAGTAGATGCCGACGCCTGATACCACATATTTTACCTGT	3′-flanking of *FpPEX11a* gene (1123bp)
a-BR	GAATTGACTCGACGCTGCAGACTC	
2-AF	gaattcggatcttccagagaCGTTATGGATAAGAGCACCGATATT	5′-flanking of *FpPEX11b* gene (1007bp)
2-AR	attcaactgccgttcgacgaGTATTGACCGATTCCTTGCGGTCCGAA	
2-BF	gaattcggatcttccagagaGATGTAGGAGGGCGTGGATATGTCCT	3′-flanking of *FpPEX11b* gene (1047bp)
2-BR	attcaactgccgttcgacgaCCGATATCGTCTTCATGTGCAG	
3-AF	gaattcggatcttccagagaGACAGGATCACTACTTAATTACCCGA	5′-flanking of *FpPEX11c* gene(1010bp)
3-AR	attcaactgccgttcgacgaGTATTGACCGATTCCTTGCGGTCCGAA	
3-BF	gaattcggatcttccagagaGATGTAGGAGGGCGTGGATATGTCCT	3′-flanking of *FpPEX11c* gene (1089bp)
3-BR	attcaactgccgttcgacgaCAGTGACAAGGACAATGCAGGTA	
aK1F	CGAGGAGCATACATATCGTGAATC	Identification of *FpPEX11a* deletion transformant 2316(bp)
K1R	ATGTTGGCGACCTCGTATTGG	
K2F	ATTAGCAGACAGGAACGAGGAC	Identification of *FpPEX11a* deletion transformant 2565(bp)
aK2R	GACGTGGTTTACTCCGAGAC	
bk1F	TCTTCTGTATCTCTGGCCACGT	Identification of *FpPEX11b* deletion transformant 2156(bp)
K1R	ATGTTGGCGACCTCGTATTGG	
K2F	ATTAGCAGACAGGAACGAGGAC	Identification of *FpPEX11b* deletion transformant 2408(bp)
bk2R	TGCTTCAACTCGCTTGGACCAG	
ck1F	TGATGAATGGTTGCCAAGTGTTTG	Identification of *FpPEX11c* deletion transformant 2054(bp)
K1R	ATGTTGGCGACCTCGTATTGG	
K2F	ATTAGCAGACAGGAACGAGGAC	Identification of *FpPEX11c* deletion transformant 2343(bp)
ck2R	CAATCGCTCTGTTCTTTGGACTG	
1-F	ATGGTCGCCGACGCAGTCAT	*FpPEX11a* fragment (761 bp)
1-R	CTAGGCGGTCTTCTTCCAC	
2-F	ATGGCTGGTACATTCGAGC	*FpPEX11b* fragment (932 bp)
2-R	CTACTGCTTCTTCATCTGAAGATCC	
3-F	CGATGTCCTCATACGCTTCCTC	*FpPEX11c*fragment (613 bp)
3-R	CGCAAACGACACCTTCATCCC	
HYG-F	GGCTTGGCTGGAGCTAGTGGAGGTCAA	The front of *hph* fragment (764 bp) H1
HY-R	GTATTGACCGATTCCTTGCGGTCCGAA	
YG-F	GATGTAGGAGGGCGTGGATATGTCCT	The later of *hph* fragment (930 bp) H2
HYG-R	AACCCGCGGTCGGCATCTACTCTATTC	
TZ-F	TCCCTATGATTGTTGTTGAACCC	*FpPEX11a* probe in Southern blot analysis (353 bp)
TZ-R	GAGACGGAGACTTGACCGAGAT	
2TZ-F	AGTCGTCTCAGCCACTTCTACCG	*FpPEX11b* probe in Southern blot analysis (373 bp)
2TZ-R	CTTGCCTTGAGATTTGGCGTTGTG	
3TZ-F	TGTGCTTGTATCAGAGGTGCCAT	*FpPEX11c* probe in Southern blot analysis(310 bp)
3TZ-R	CCGTTAGAGGTTTGTTTGAGCC	
TUB-F	CTTACGGCGACCTGAACTACCTTG	qRT-PCR analysis

TUB-R	AGCGAATCCGACCATGAAGAAGTG
Tri5-F	GAGTGTTTCATGCATGGCTACGTC
Tri5-R	CTGAGCCTCCTTCACATCGTCC
Tri6-F	CTGAGGGCATTCTGAGTAGCGACA
Tri6-R	CGTTATGTTTATCGGCACTTTG
Tri10-F	GCGACAGGAGCAAGAACATAA
Tri10-R	GGCGGCGTAAATCTGAGTG
PTH2-F	AGAACCACTCAAAGGGACCAATGC
PTH2-R	TAGATAACGGCTGGCTGTCTCCTC
DNM1-F	GATGTCGTCCAGAACCGTCTTGTC
DNM1-R	CTCGCACTTCTCACGCTCCTTC
PEX3-F	GAGCAGTGATCGTATCGCCAAGG
PEX3-R	CGAGTGTGATCCGTTCCGTGTTC
PEX19-F	GCTCCTACGCATTACCAAGTCTCG
PEX19-R	CATCCATCAGCTCGCTCTCACTTC
PEX11a-F	GAGGGAGAGGGCGTCGTTGAG
PEX11a-R	ATATCGCAGACATCGCAGAGAAGC
PEX11b-F	CCTCTTGGATCTCTGCGGCATTG
PEX11b-R	ACAGCCCAGCAAACCAGAACAG
PEX11c -F	TTGCCGCACGGAGCTTAACG
PEX11c-R	GGGGACGAAACCGATGCTATTGAG
FIS1-F	CGGCGTCCAGACCAAGTTCAAC
FIS1-R	AGGGCGAGGTAGTAGAGGCATTC
PIP2-F	TGACAAGCGGTTGAAGCGTATGG
PIP2-R	AGTGACGGACGATGCGACCTC
